# Catecholamines Are the Key Trigger of COVID-19 mRNA Vaccine-Induced Myocarditis: A Compelling Hypothesis Supported by Epidemiological, Anatomopathological, Molecular, and Physiological Findings

**DOI:** 10.7759/cureus.27883

**Published:** 2022-08-11

**Authors:** Flavio A Cadegiani

**Affiliations:** 1 Clinical Endocrinology, Corpometria Institute, Brasilia, BRA; 2 Clinical Endocrinology, Applied Biology, Inc., Irvine, USA

**Keywords:** hypercatecholaminergic, myocarditis, sudden death, athlete, catecholamine, sars-cov-2 spike protein, sars-cov-2 mrna vaccine, sars-cov-2, covid-19

## Abstract

Severe acute respiratory syndrome coronavirus 2 (SARS-CoV-2) mRNA vaccine-induced myocarditis is a rare but well-documented complication in young males. The increased incidence of sudden death among athletes following vaccination has been reported and requires further investigation. Whether the risk of myocarditis, a known major cause of sudden death in young male athletes, also increases after coronavirus disease 2019 (COVID-19) infection is unknown. The severity and implications of these critical adverse effects require a thorough analysis to elucidate their key triggering mechanisms. The present review aimed to evaluate whether there is a justification to hypothesize that catecholamines in a “hypercatecholaminergic” state are the key trigger of SARS-CoV-2 mRNA vaccine-induced myocarditis and related outcomes and whether similar risks are also present following COVID-19 infection.

A thorough, structured scoping review of the literature was performed to build the hypothesis through three pillars: detection of myocarditis risk, potential alterations and abnormalities identified after SARS-CoV-2 mRNA vaccination or COVID-19 infection and consequent events, and physiological characteristics of the most affected population. The following terms were searched in indexed and non-indexed peer review articles and recent preprints (<12 months): agent, “SARS-CoV-2” or “COVID-19”; event, “myocarditis” or “sudden death(s)” or “myocarditis+sudden death(s)” or “cardiac event(s)”; underlying cause, “mRNA” or “spike protein” or “infection” or “vaccine”; proposed trigger, “catecholamine(s)” or “adrenaline” or “epinephrine” or “noradrenaline” or “norepinephrine” or “testosterone”; and affected population, “young male(s)” or “athlete(s).” The rationale and data that supported the hypothesis were as follows: SARS-CoV-2 mRNA vaccine-induced myocarditis primarily affected young males, while the risk was not observed following COVID-19 infection; independent autopsies or biopsies of patients who presented post-SARS-CoV-2 mRNA vaccine myocarditis in different geographical regions enabled the conclusion that a primary hypercatecholaminergic state was the key trigger of these events; SARS-CoV-2 mRNA was densely present, and SARS-CoV-2 spike protein was progressively produced in adrenal medulla chromaffin cells, which are responsible for catecholamine production; the dihydroxyphenylalanine decarboxylase enzyme that converts dopamine into noradrenaline was overexpressed in the presence of SARS-CoV-2 mRNA, leading to enhanced noradrenaline activity; catecholamine responses were physiologically higher in young adults and males than in other populations; catecholamine responses and resting catecholamine production were higher in male athletes than in non-athletes; catecholamine responses to stress and its sensitivity were enhanced in the presence of androgens; and catecholamine expressions in young male athletes were already high at baseline, were higher following vaccination, and were higher than those in non-vaccinated athletes.

The epidemiological, autopsy, molecular, and physiological findings unanimously and strongly suggest that a hypercatecholaminergic state is the critical trigger of the rare cases of myocarditis due to components from SARS-CoV-2, potentially increasing sudden deaths among elite male athletes.

## Introduction and background

Coronavirus disease 2019 (COVID-19), a multisystemic disease caused by the severe acute respiratory syndrome coronavirus 2 (SARS-CoV-2), was first detected in Wuhan, China, at the end of 2019. COVID-19 led to the first pandemic of the 21st century, causing more than six million deaths and 800 million cases until June 2022.

The peculiarities of SARS-CoV-2 caused a disease characterized by complex pathophysiology that has not been fully elucidated and changes across its variants, leading to widely varying disease presentation and severity. This indicates that COVID-19 depends more on the host (humans) than on the virus itself. This particularity of COVID-19 also implies that the monitoring of COVID-19 effects should be age-, sex-, and comorbidity-specific for a more precise understanding of the risk factors not only for COVID-19 directly related outcomes but also for further complications.

As the large variability in presentation, severity, and complications observed in COVID-19 is likely attributed to the specificities of SARS-CoV-2 mRNA and spike protein, it should be expected and therefore presumed that SARS-CoV-2 vaccines of any type, which expose individuals to these proteins, should also be monitored in a characteristic-specific manner.

Surveillance of long-term effects of COVID-19 and SARS-CoV-2 vaccines

Efforts to mitigate the COVID-19 pandemic have been tremendous and included multiple public health recommendations and mandates, pharmacological treatments, and vaccine development. Vaccines of different types, including attenuated virus, viral vector, and SARS-CoV-2 mRNA vaccines, were developed at record speed to protect humans against COVID-19 and its outcomes. Despite regional disparities, the massive worldwide vaccination for COVID-19 achieved in <1 year may have contributed to an overall reduction in COVID-19 cases and, to a greater extent, COVID-19 deaths.

However, neither the characterization of COVID-19’s long-term effects, including post-COVID-19 or long COVID-19 or increased complications after COVID-19, nor the long-term safety profile and less common adverse effects of SARS-CoV-2 vaccines is well established. Ethically, collective thorough surveillance of complications after COVID-19 and SARS-CoV-2 vaccination is mandatory. Correspondingly, every event chronologically related to COVID-19 infection or SARS-CoV-2 vaccination should be presumably considered as being related until proved otherwise. When the incidence of a certain event is apparently higher after COVID-19 infection or SARS-CoV-2 vaccination within a population of a specific sex and age range when compared to the incidence of this event in the same population before the COVID-19 pandemic or the start of COVID-19 vaccination or to the same population without exposure to SARS-CoV-2 or SARS-CoV-2 vaccines, further investigations must be conducted to fully elucidate the event. Active surveillance and investigations should be equally used for events related to COVID-19 infection and SARS-CoV-2 vaccines non-discriminatorily.

Despite insufficient surveillance, some post-SARS-CoV-2 infection and post-SARS-CoV-2 vaccination effects have been identified. An increased number of reports of myocarditis in young males and sudden deaths in male athletes chronologically following SARS-CoV-2 mRNA vaccination raised concern and led to initial investigations regarding the correlation and causality between these events and this specific type of SARS-CoV-2 vaccine. The evidence indicating that cardiac risks are specific to SARS-CoV-2 mRNA vaccines rather than to COVID-19 or post-COVID is compelling, as multiple independent groups and data from different regions demonstrated not only similar findings but also similar patterns that the second SARS-CoV-2 mRNA vaccine dose presents a stronger risk of myocarditis and pericarditis than the first dose and that the young population, particularly males, are at the highest risk. Conversely, despite the overwhelming increase in reports of sudden deaths in male athletes and young males, there remains a lack of more robust literature to support this claim.

Myocarditis and sudden death after COVID-19 mRNA vaccination

Myocarditis induced by SARS-CoV-2 mRNA vaccines is an indisputable complication observed particularly in young males [1-4] as demonstrated by multiple studies in different populations [1-11]. Indeed, two studies reported that COVID-19 mRNA vaccine-induced myocarditis disproportionately affected adolescents (reporting odds ratio (ROR): 22.3; 95% confidence interval (CI): 19.2-25.9), 18-29-year-olds (ROR: 6.6; 95% CI: 5.9-7.5), and males (ROR: 9.4; 95% CI: 8.3-10.6) [5,6]. These findings were supported by another large registry study that identified increased myocarditis risk following SARS-CoV-2 mRNA vaccination, with the highest risk detected in people aged 18-24 years, particularly after the second dose, where 8.1-fold increased risk after the BNT162b2 SARS-CoV-2 mRNA vaccine (95% CI: 6.7-9.9) and 30-fold increased risk after the mRNA-1273 SARS-CoV-2 vaccine (95% CI: 21-43) were reported [7]. An Israeli government dataset demonstrated 13.6-fold increased myocarditis risk (95% CI: 9.3-19.2) among males aged between 16 and 19 years compared to the expected following historical data, while a ninefold increased myocarditis risk (95% CI: 4.5-17.8) was recorded when compared to unvaccinated people of similar age and sex during the same period [8]. In a 23 million-resident area, the myocarditis risk after SARS-CoV-2 mRNA vaccination was increased across all populations but was particularly high among males aged 16-24 years after the second dose of BNT162b2 and mRNA-1273, where 5.3-fold (95% CI: 3.7-7.7) and 13.8-fold (95% CI: 8.1-23.7) increased myocarditis risk was recorded, respectively [9].

Among all population studies that universally detected increased myocarditis risk after SARS-CoV-2 mRNA vaccination, a nationwide fully controlled Israeli dataset identified a strict correlation between both first and second SARS-CoV-2 mRNA vaccine doses and an increase in emergency calls, particularly in those between the ages of 16 and 39 years, after adjustments for confounders [10]. The fact that two peaks were observed chronologically following the two SARS-CoV-2 mRNA vaccine doses strongly reinforced the correlation.

Not only were myocarditis risks identified, but a great burden following these cardiac events was also described [7]. Indeed, young males with comorbidities may experience dramatic cardiac remodeling following SARS-CoV-2 mRNA vaccination [11].

Although preliminary comparisons between COVID-19 infection and SARS-CoV-2 mRNA vaccines demonstrated similar increased myocarditis risk in athletes and people aged <40 years [12-14], the risk of consequential heart arrhythmia was significantly higher after vaccination than after COVID-19 [12], possibly supporting the apparent higher incidence of sudden deaths after COVID-19 vaccination than after COVID-19. Indeed, myocarditis was only detected through active biochemical surveillance monitoring of post-COVID-19 athletes [13,14], which has not been used in athletes after SARS-CoV-2 mRNA vaccination. This is justifiable as more active follow-up after a symptomatic disease is expected rather than after a vaccine in otherwise healthy and asymptomatic people.

Indeed, the alleged similar increase in myocarditis risk following COVID-19 infection and SARS-CoV-2 mRNA vaccines was refuted. While findings of overwhelming increases in symptomatic myocarditis have been extensively demonstrated and reproduced, another strictly controlled Israeli dataset involving a large population study with 196,992 COVID-19 cases and 590,976 controls demonstrated similar myocarditis incidence between people who had been infected with COVID-19 and among non-COVID-19-infected patients [15].

Correspondingly, although an increase in sudden deaths among young people has been reported since the 1990s [16], the incidence of sudden deaths among athletes appears to have increased sharply in 2021 [17]. Chronologically, this coincides with the increased proportion of vaccinated athletes, with far more males affected than females, and the increase was particularly high in 2021 [5].

However, this is an anecdotal and empirical observation that has not been confirmed, while numbers are highly underestimated [18], obscuring a sudden death outbreak among athletes. Although preliminary and unofficial, specific data have been reported among professional male soccer players. The number of sudden deaths during soccer training among the International Federation of Association Football (FIFA) professional soccer players ranged between five and 10 deaths yearly between 2009 and 2019, and only two were reported in 2020; there was a yearly average of 7.8 deaths between 2009 and 2020. In 2021, 31 sudden deaths were reported [17,19,20].

Correlation between myocarditis and sudden death

The correlations between myocarditis and sudden death vary widely according to the population studied [21]. However, it is accepted that a non-negligible percentage of sudden deaths occur due to myocarditis. Myocarditis is a major cause of sudden death among young people with an incidence that is twice as high in males than in females and is even more prevalent among athletes [21-23].

Hypothesis and objective

While young males and male athletes appear to be at higher risk of cardiac events as observed in a chronologically correlative manner, causality has not been established. The particularities of these populations should be prioritized as potential causes. The two major aspects hypothesized to be specific to these populations are high testosterone and high catecholamine (noradrenaline and adrenaline) levels, which alone or combined may experience interference by SARS-CoV-2 components, including the disease (COVID-19) and vaccines. In particular, there appear to be overwhelming chronological coincidences between catecholamine peaks and the incidence of the associated cardiac events. From these observations, it was hypothesized that a “hypercatecholaminergic” state, increased catecholamine levels, and enhanced sensitivity to catecholamines combined with high testosterone levels may mediate SARS-CoV-2 mRNA vaccine-induced myocarditis and related events. In the present article, a scoping review of the emerging literature was performed to substantiate or refute the proposed hypothesis. Ultimately, this article does not aim to discourage or stimulate hesitation regarding SARS-CoV-2 vaccines but to detect and propose explanations for the phenomenon, leading to more effective monitoring and prevention of further events.

Materials and methods

Are catecholamines under a hypercatecholaminergic state the key trigger of COVID-19 mRNA vaccine-induced myocarditis and related sudden deaths?

While the incidence of sudden deaths among athletes is uncertain, the occurrence of COVID-19 mRNA vaccine-induced clinically detectable myocarditis is unquestionable. The underlying mechanisms of both potentially linked situations deserve further elucidation. For the reasons described in the following paragraphs and through extensive literature research, evaluation was performed on the proposed hypothesis: catecholamines under a hypercatecholaminergic state that includes enhanced catecholamine responses, sensitivity, and action might be the key mechanism for inducing myocarditis and consequent sudden death, particularly in young males and athletes.

To build the hypothesis, the following pillars for the hypothesis must be characterized: detection of the risk of the event (myocarditis and related outcomes) (pillar 1) (for detecting the phenomenon), and potential alterations and abnormalities identified after SARS-CoV-2 mRNA vaccination or COVID-19 infection and consequent events (pillar 2) and physiological characteristics specific to the population likely most affected by myocarditis and the related outcomes (pillar 3) (for searching for the etiology of the phenomenon).

To characterize each pillar, the following specific questions should be answered. For pillar 1, the questions are as follows: (1) Do SARS-CoV-2 mRNA vaccines lead to increased myocarditis risk? If so, are young males and male athletes at the highest risk for this complication? Is the risk dose-dependent, i.e., do further doses lead to further increments in myocarditis risk? (2) Does this also apply to pericarditis, cardiac events, and sudden deaths? (3) Is the same increased myocarditis risk also observed during or after COVID-19 infection? If so, do the characteristics of this event have similar severity, prognosis, and population affected as the events after SARS-CoV-2 mRNA vaccination?

The questions for pillar 2 are as follows: (1) Does any between SARS-CoV-2 mRNA vaccines or COVID-19 infection lead to increased catecholaminergic (noradrenaline or adrenaline) activity at any level, including increased secretion from the adrenal medulla and sympathetic ganglions, increased catecholamine receptor sensitivity, or increased response to catecholamine signaling? In summary, do any of them lead to a hypercatecholaminergic state? (2) Has it been demonstrated that any of the specific populations that presented the highest myocarditis risk also presented increased catecholaminergic activity with SARS-CoV-2 mRNA vaccines? (3) Are myocarditis and the related outcomes influenced or induced by a hypercatecholaminergic state?

For pillar 3, the questions are as follows: (1) Do young males present increased catecholaminergic activity of any sort compared to older males or to females? (2) Do male athletes present increased catecholaminergic activity of any sort compared to sex- and age-matched non-athletes? (3) Do these two populations of young males and male athletes present higher testosterone levels compared to other populations? (4) If young males, male athletes, or both present both increased catecholaminergic activity and testosterone levels, do these two particularities play a synergistic role leading to further enhancement of the activity of these hormones? In this specific case, do higher testosterone levels lead to further increases in catecholaminergic activity?

Once these three pillars are well-characterized, the hypothesis that a hypercatecholaminergic state is likely a key trigger of SARS-CoV-2 mRNA vaccine-induced myocarditis can be confirmed, reinforced, undermined, or refuted.

The detection of the phenomenon, i.e., the answers to the questions regarding pillar 1, has been answered in the Introduction section and forms the background of the proposed hypothesis.

The answer to pillar 1 is that increased myocarditis risk has been extensively confirmed after SARS-CoV-2 mRNA vaccination, while it is unlikely to be present following COVID-19 infection. The increased myocarditis risk detected in young males was highest (6-30-fold increased risk) as compared to that in unvaccinated age- and sex-matched controls or to myocarditis incidence in the same population before COVID-19 mRNA vaccination.

Whether myocarditis risk is even higher among athletes (particularly male athletes) and whether the incidence of sudden death (or not) following myocarditis also increases after SARS-CoV-2 mRNA vaccination are less clear but deserve consideration for the present hypothesis. Under uncertainties, as the principle of caution should prevail under uncertainties, these events must be considered real risks until demonstrated otherwise.

While the questions for pillar 1 were answered in the Introduction and formed the rationale for the proposed hypothesis, the answers to the questions for pillars 2 and 3 constitute the objective of the present analysis, which will be performed in the Results section following the search methods described below.

Design and search methods

A scoping review of the existing literature was performed and applied to the hypothesis development process. The corresponding literature in indexed peer-reviewed articles in PubMed (MEDLINE), indexed and non-indexed peer-reviewed articles and preprints on Google Scholar, and recent preprints (after July 1, 2021) in medRxiv, Research Square, and ResearchGate were searched. Expressions regarding the following were searched: agent, “SARS-CoV-2” or “COVID-19”; event, “myocarditis” or “sudden death(s)” or “myocarditis+sudden death(s)” or “cardiac event(s)”; underlying cause, “mRNA” or “spike protein” or “infection” or “vaccine”; proposed trigger, “catecholamine(s)” or “adrenaline” or “epinephrine” or “noradrenaline” or “norepinephrine” or “testosterone”; and affected population, “young male(s)” or “athlete(s).”

**Figure 1:**
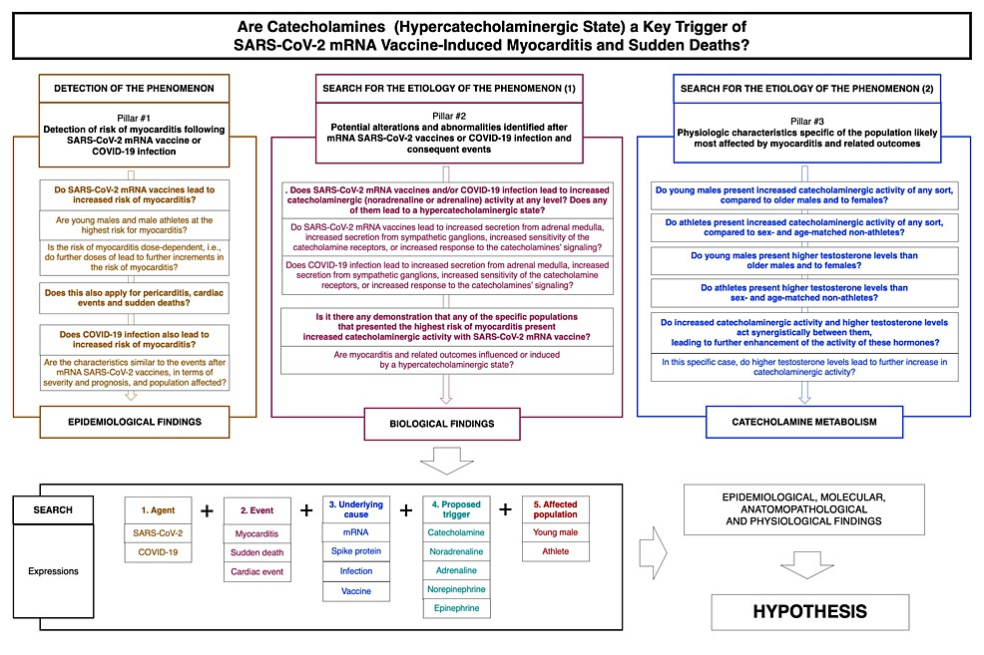
Pillars and search methods for the proposed hypothesis.

If the description of the phenomenon potentially linked with SARS-CoV-2 components or COVID-19 was recent, the search was not restricted to published articles. As this is a descriptive review aiming to describe a hypothesis, a systematic review, although structured, was therefore not the objective of the present article. Figure 1 depicts the pillars for the proposed hypothesis and the expressions searched to build the hypothesis.

## Review

Findings from autopsies of post-COVID-19 mRNA vaccination deaths

Autopsies were performed on two adolescents who died from myocarditis three and four days after BNT162b2 vaccination. Both cases were negative for medication or drug misuse. Histologically, the cardiomyocytes presented extensive diffuse contraction band necrosis (CBN) with hypercontracted sarcomeres and mononuclear inflammatory response. These findings contrast those following infarction, in which polymorphonuclear inflammation is observed [24].

The autopsy of a 22-year-old male after sudden death due to myocarditis a few days after the first dose of the BNT162b2 vaccine revealed no inflammatory or cellular response or thrombosis in the ventricular area. Conversely, cardiomyocyte necrosis and scattered single-cell necrosis, degeneration, and adjacent inflammatory infiltrate were noted. The most remarkable characteristic was multiple scattered foci of CBN throughout the myocardium predominantly in the left ventricle, which is the most muscular heart chamber. These findings define catecholamine-induced myocarditis, mixing the early (necrotic and damaged cardiomyocytes) and later (inflammation) stages of injury caused by catecholamines [25].

In both reports, the possibility of any hypersensitivity reaction was excluded as eosinophilic infiltrate was absent. Overall, the injury pattern in all three cases was consistent with stress cardiomyopathy with contraction bands caused by excessive catecholaminergic activity (i.e., hyperadrenergic, “hypernoradrenergic,” or both). These histopathological findings are exceedingly similar to the non-rare myocarditis complication of pheochromocytoma, a catecholamine-producing tumor in the adrenal medulla due to abnormal chromaffin cell proliferation [26-28].

Before the specific characteristics of catecholamine-induced myocarditis were searched, two cases from among the first reported cases of myocarditis following SARS-CoV-2 mRNA vaccination demonstrated indirect signals of a hypercatecholaminergic state [29].

The first case was a 45-year-old female who presented acute heart failure due to fulminant myocarditis 10 days after the first dose of the BNT162b2 vaccine. All other causes that could potentially lead to myocarditis, including COVID-19, coronary artery disease, other infections, and other inflammatory or autoimmune diseases, were excluded. The standard therapy for congestive heart failure (metoprolol, lisinopril, and spironolactone) led to an unexpectedly sharp rise in the left ventricle ejection fraction within seven days of therapy from 15%-20% to 60%, a faster recovery than expected. The fast recovery may solely be explained if the underlying triggering cause of the myocarditis had been obstructed. In the present case, the direct antagonist actions of a potential hyperadrenergic state that may have led to the myocarditis state with a beta-adrenergic blocker (metoprolol) may have justified the prompt recovery, although further actions of the angiotensin-converting enzyme inhibitor (ACEi) (lisinopril) and mineralocorticoid antagonist (spironolactone) may have exerted synergistic protective effects on ACE expression and balance between its receptors.

The second case was a 42-year-old male who died after cardiac shock caused by myocarditis, which clinically manifested as fever, tachycardia, dyspnea, and chest pain, that started two weeks after a second dose of the mRNA-1273 vaccine. Other causes of myocarditis and death, including COVID-19, other viral infections, and obstructive coronary artery disease, were excluded. Similar to the first case, this fatal event was largely attributed to a hypercatecholaminergic state, which was unlikely to be secondary to infections or any type of inflammation-induced diseases as these conditions had been excluded. This reinforced the hypothesis of a primary hypercatecholaminergic state as a trigger of this event.

The first report of severe acute myocarditis after the third dose of the SARS-CoV-2 mRNA vaccine was in a 43-year-old female who presented rapid monomorphic ventricular tachycardia (VT) potentially caused by a catecholaminergic storm. For myocarditis and VT, diagnoses of acute coronary disease, COVID-19, other viral or bacterial infections, autoimmune disorders, or any other disease known to cause myocarditis and VT were excluded [30]. Despite glucocorticoid, ACEi, and beta-blocker treatment, the patient experienced a persistent recurrent monomorphic VT episode two weeks after the first event and required an increased beta-blocker dose. The increased blockage of the adrenergic actions appeared to lead to complete resolution of the myocarditis.

In all three cases, CD3+ T lymphocytes and CD20+ B lymphocytes were highly present, while macrophages, eosinophils, and other immune cells were almost absent, indicating a lymphocyte-specific induced inflammatory state. These findings supported the hypothesis that all three cases may have been triggered by a hypercatecholaminergic state, as adrenaline and noradrenaline tend to activate lymphocytes while inhibiting monocytes, macrophages, and eosinophils [31,32].

Unlike cases of post-SARS-CoV-2 mRNA vaccine myocarditis, the autopsies from deaths due to cardiovascular events after COVID-19 infection and other types of SARS-CoV-2 vaccine did not demonstrate contraction bands in the cardiomyocytes or any other indication of damage caused by excessive catecholaminergic activity in any tissue [33], demonstrating the specificness of the damage caused by SARS-CoV-2 mRNA vaccines.

The bridge between myocarditis and sudden death after COVID-19 mRNA vaccination: a hyperadrenergic state

Autopsies have demonstrated that catecholamines triggered myocarditis following COVID-19 mRNA vaccination. The bridge connecting myocarditis and sudden death is found in the chronic hyperadrenergic state, a plausible and demonstrated cause [21-23]. Excessive long-term adrenaline and noradrenaline release, particularly at rest, is an independent predictor of sudden death [34,35].

The adrenal glands have been repeatedly demonstrated to be a major site of SARS-CoV-2 mRNA accumulation and SARS-CoV-2 spike protein production, indicating that both the adrenal cortex and medulla were affected. A report evaluating spike protein production after COVID-19 mRNA vaccination determined that the adrenal glands were one of the highest SARS-CoV-2 spike protein-producing tissues, demonstrating that the spike protein production in these glands increased with time [36]. Furthermore, notable, robust, and dense SARS-CoV-2 RNA expression and spike protein presence were detected in the adrenal medulla of animal models and the overall adrenal glands in humans [37]. Although focal or diffuse inflammation and swollen and necrotic chromaffin cells were noted in the adrenal medulla, the adrenal cortex might also have been affected [38].

Despite the lack of a direct comparison, post-COVID-19 autopsies demonstrated that while SARS-CoV-2 mRNA was detected diffusely, including in the adrenal glands [39], its concentration in these glands was not as specific as that described after COVID-19 mRNA vaccination.

That high SARS-CoV-2 mRNA concentration and corresponding abnormalities in the chromaffin cells that could lead to a catecholamine storm was not the only demonstrated finding. The enzyme dihydroxyphenylalanine (DOPA) decarboxylase converts DOPA into dopamine, the precursor of noradrenaline (through dopamine-beta-hydroxylase) and adrenaline (converted from noradrenaline by phenylethanolamine-N-methyltransferase (PNMT)). DOPA decarboxylase is overexpressed in the presence of SARS-CoV-2 mRNA, a phenomenon not restricted to the adrenal glands. Such overexpression does not occur with other viruses, such as influenza. Naturally, an enhanced diffused conversion from dopamine to noradrenaline occurs locally in the tissues, disproportionately increasing their exposure to catecholamines compared to their serum levels. In addition to the potentially enhanced catecholamine production and release, and beta-2 adrenergic receptor hypersensitivity in tissue, disproportionally high adrenaline levels might be present [40].

Physiological differences in catecholamine metabolism according to age, sex, and activity: do young males and athletes have the highest hyperadrenergic activity?

Age, sex, and athletic-level physical activity present pronounced differences in the physiology of catecholamine metabolism. Athletes, particularly male athletes, had significantly higher accumulated levels of active catecholamines, even at rest, compared to non-athletes. This observation was based on measuring 12-hour nocturnal urinary catecholamines, which refers to the accumulated release of these hormones throughout the night. While athletes had higher catecholamine levels than sex-, age-, and comorbidity-matched non-athletes, the catecholamine metabolite metanephrine remained unchanged, suggesting unaltered catecholamine clearance that led to a chronic physiological increase in exposure [41-43].

While athletes present higher catecholamine release and responsiveness than non-athletes, virtually, all people analyzed in these studies were aged <40 years, i.e., age was not a major aspect considered in these analyses. Older adults tend to present lower catecholamine activity than young people. A significant age-related decrease in noradrenaline expression and uptake was observed in rats [44]. The decrease with age was specifically noted in the chronotropic, ionotropic, and biochemical responses of the beta-adrenergic receptor in cardiomyocytes in both animal and human models [45,46]. The only responses in catecholamine metabolism seemingly unaffected by aging were the medullary adrenal responses to stress [47].

It is well-established that males present higher catecholamine responses to various stress types than females [48-50]. Moreover, both catecholamine release and beta-2 adrenergic receptor responsiveness in the cardiomyocytes are more pronounced in males than in females [51].

Although the association between sex and the age-related decrease in catecholamine activity was not reported, the age-related and sex-specific catecholamine responses and release might reflect an important interplay between catecholamines and androgens. Catecholamine responses to stress and adrenergic receptor sensitivity are enhanced in the presence of androgens [52,53]. This association with androgens is concordant with the occurrence of COVID-19 mRNA vaccine-induced myocarditis and sudden death predominantly in young males [1-5,17-20].

Dysfunctional, exacerbated catecholamine metabolism in young males and athletes after COVID-19 mRNA vaccination

Already physiologically exposed to high catecholamine levels, athletes experience additional exposure after COVID-19 mRNA vaccination. Although underestimated, recently vaccinated athletes presented significantly higher noradrenaline production in response to graded exercise than before vaccination (p < 0.01). The researchers also compared participants vaccinated with the Pfizer mRNA vaccine and non-vaccinated participants and demonstrated higher noradrenaline production (p = 0.04 at 80% maximal oxygen consumption (VO2max)) and heart rate (p = 0.006 at 70% VO2max). All participants underwent a similar graded exercise program controlled through respiratory parameters [54].

Myocarditis and sudden death are highly specific to young males, particularly male athletes, the population with the highest catecholamine exposure. The underlying cause of these two severe outcomes is a common hyperadrenergic state, universally demonstrated through autopsies that concluded adrenaline-induced myocarditis. Therefore, the plausibility of the hypothesis that catecholamines are the key to the occurrence of myocarditis and sudden death after COVID-19 mRNA vaccinations is considerable and is most likely but requires molecular confirmation.

Further hypothesis-supporting data

The physiological differences in catecholamine responses and the dysfunctional changes in catecholamine production and action after COVID-19 mRNA vaccination strongly support the proposed hypothesis.

Local paracrine-acting catecholamine release in response to stress in various tissues is directly related to the adrenal noradrenaline output level. This means that the already physiologically high catecholamine responses observed in young male athletes undergo additional enhancements after COVID-19 mRNA vaccination. These paracrine effects might become even more pronounced upon analysis of the tissue catecholamine concentration and action in various sites, particularly the vessels and muscles, including the cardiomyocytes [55].

A retrospective analysis demonstrated that the use of alpha-1 adrenergic receptor antagonists reduced COVID-19 in-hospital mortality by 77% (odds ratio (OR): 0.23; 95% CI: 0.03-0.94; p = 0.028) [56], while beta-blockers reduced the inflammatory response that led to cytokine storms in COVID-19 [57]. In addition, SARS-CoV-2 mRNA vaccination has been followed by multiple cases of Takotsubo cardiomyopathy [58], a syndrome triggered by excessive catecholamine release and activity [59].

These findings demonstrate the pro-inflammatory effects, harm, and consequent outcomes caused by the excessive catecholaminergic state due to SARS-CoV-2 mRNA and/or spike protein [60], thereby supporting the proposed hypothesis that catecholamines play an important role in myocarditis induced by SARS-CoV-2 mRNA and/or spike protein.

Conversely, the cause of sudden deaths among athletes was largely attributed to ventricular tachycardia following a hypercatecholaminergic state, which may have also induced concurrent myocarditis that could together lead to VT [61,62]. Both the etiology of sudden death in athletes and the main trigger of myocarditis identified after SARS-CoV-2 mRNA vaccination converge on hypercatecholaminergic activity as the common cause, providing further confirmation of the proposed hypothesis.

**Figure 2:**
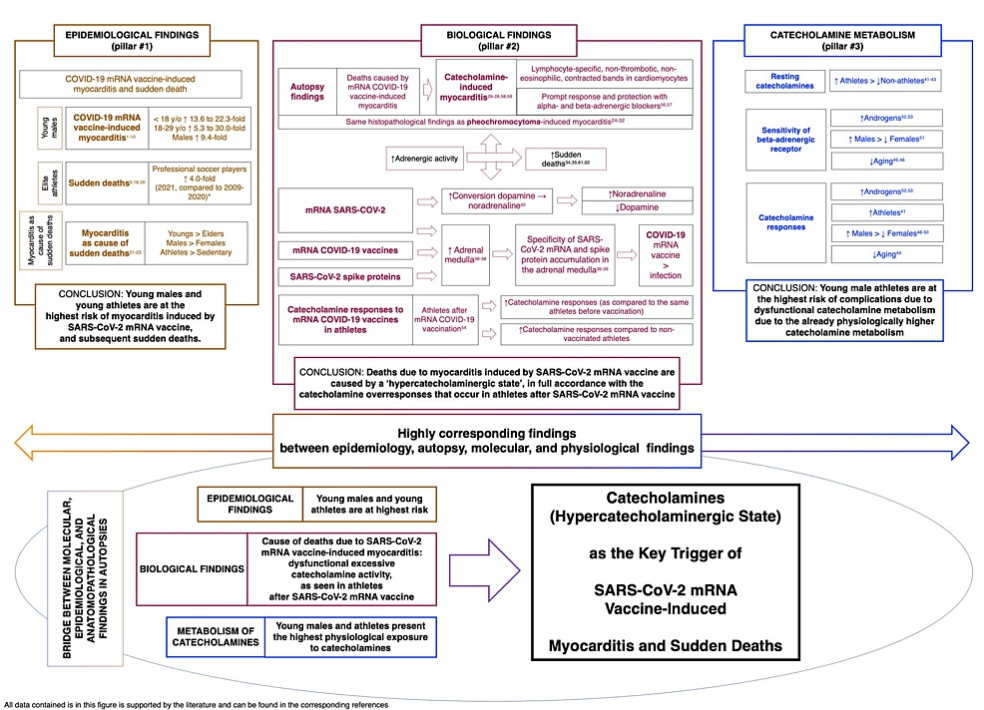
Summary of the rationale and supporting data for the present hypothesis.

Figure 2 summarizes the findings that support the hypothesis of a hypercatecholaminergic state being a key trigger of myocarditis and consequent events induced by SARS-CoV-2 mRNA vaccines, which include epidemiological and biological findings and the peculiarities of catecholamine metabolism in the most affected population.

Discussion

Synthesizing Epidemiological, Anatomopathological, Molecular, and Physiological Findings to Propose a Hypothesis of Catecholamines as the Key Trigger of SARS-CoV-2 mRNA Vaccination-Induced Myocarditis and Sudden Death

Altogether, the evidence included the following: the epidemiological findings of COVID-19 mRNA vaccine-induced myocarditis and the potential increase in sudden deaths among young males, particularly athletes; anatomopathological findings during autopsies of myocardial tissue that demonstrated a clear state of catecholamine-triggered myocarditis in all cases; the notable presence of SARS-CoV-2 mRNA and enhanced SARS-CoV-2 spike protein production in adrenal medulla chromaffin cells; the overexpression of the DOPA decarboxylase enzyme in the presence of SARS-CoV-2 mRNA, leading to enhanced conversion of dopamine into noradrenaline; higher physiological catecholamine metabolism in younger people compared to older people, males more than females reinforced by the positive catecholamine response and sensitivity in the presence of androgens, and athletes more than non-athletes where the former is likely the most affected population; and more enhanced catecholamine response in vaccinated athletes than non-vaccinated or pre-vaccinated athletes. All this evidence was fully concordant, which supported the proposed hypothesis that catecholamines are a key player in the SARS-CoV-2 mRNA vaccine-induced myocarditis and the consequent apparent increase in sudden deaths.

It is unlikely that the enhanced catecholamine release, response, receptor sensitivity, and overall activity acted alone to provoke the vaccine-induced, catecholamine-triggered myocardial complications. The catecholamines possibly acted synergistically with other dysfunctions, including abnormal immunological and inflammatory responses, as they alone may cause myocarditis only during extreme catecholamine exposure.

It remains unclear whether the proteins transcribed by SARS-CoV-2 mRNA or SARS-CoV-2 spike protein or both trigger the hypercatecholaminergic state that eventually causes myocarditis after SARS-CoV-2 mRNA vaccination. However, this should be explored in further study after the proposed hypothesis has been confirmed.

## Conclusions

The epidemiological findings of SARS-CoV-2 mRNA vaccine-induced myocarditis, which is overrepresented in young males, and the preliminary reports of an increase in sudden deaths, particularly in athletes, while absent after COVID-19 infection present highly concordant molecular justifications for the physiological differences in anatomopathological findings and catecholamine activity, which is more intense in active young males. We may conclude that supported by biological, clinical, and epidemiological findings, enhanced catecholamine activity or a hypercatecholaminergic state provides sufficient evidence for the highly plausible catecholamine theory of SARS-CoV-2 mRNA or spike protein-mediated myocardial complications to be considered a strong hypothesis.
